# Numerical Investigation of Galloping Instabilities in Z-Shaped Profiles

**DOI:** 10.1155/2014/363274

**Published:** 2014-06-25

**Authors:** Ignacio Gomez, Miguel Chavez, Gustavo Alonso, Eusebio Valero

**Affiliations:** ^1^E.T.S.I. Aeronáuticos, Universidad Politécnica de Madrid, 28040 Madrid, Spain; ^2^IDR/UPM, E.T.S.I. Aeronáuticos, Universidad Politécnica de Madrid, 28040 Madrid, Spain

## Abstract

Aeroelastic effects are relatively common in the design of modern civil constructions such as office blocks, airport terminal buildings, and factories. Typical flexible structures exposed to the action of wind are shading devices, normally slats or louvers. A typical cross-section for such elements is a Z-shaped profile, made out of a central web and two-side wings. Galloping instabilities are often determined in practice using the Glauert-Den Hartog criterion. This criterion relies on accurate predictions of the dependence of the aerodynamic force coefficients with the angle of attack. The results of a parametric analysis based on a numerical analysis and performed on different Z-shaped louvers to determine translational galloping instability regions are presented in this paper. These numerical analysis results have been validated with a parametric analysis of Z-shaped profiles based on static wind tunnel tests. In order to perform this validation, the DLR TAU Code, which is a standard code within the European aeronautical industry, has been used. This study highlights the focus on the numerical prediction of the effect of galloping, which is shown in a visible way, through stability maps. Comparisons between numerical and experimental data are presented with respect to various meshes and turbulence models.

## 1. Introduction

Aeroelastic phenomena are becoming more and more important from the point of view of its potential relevance in modern structural design. It is well known that bluff bodies in cross-flow are subject to typical aeroelastic phenomena like vortex shedding, translational and torsional galloping, and even flutter. Some of these phenomena can even appear coupled occasionally. Galloping is a typical instability of flexible, lightly damped structures. Under certain conditions, these structures may have large amplitude, normal to wind oscillations, at much lower frequencies than those of vortex shedding found in the von Kármán vortex street.

Theoretical foundations of galloping are well established and understood [1], very often supported by experiments, like in Parkinson and Smith [2], Novak [3], Novak [4], and Simiu and Scanlan [5]. As it is well known, galloping can be explained by taking into account that although the incident velocity *U* is uniform and constant, because of the lateral oscillation of the body, in a body reference system, the total velocity changes both magnitude and direction with time. Therefore, the structure angle of attack also changes with time, hence the aerodynamic forces acting on the body.

In the simplest model of galloping (one degree of freedom model), it is assumed that the two-dimensional body (*x*-*z* plane), whose mass per unit length is *m*, is elastically mounted on a support with a damping coefficient *ζ* and a stiffness *mω*_*n*_^2^ (where *ω*_*n*_ is the angular natural frequency). This structure at rest is oriented at given angle of attack *α*_0_ with respect to the incident flow. Assuming the structure oscillating along *z*-axis direction within an uniform flow with velocity *U*, the relative velocity between the fluid and the body is *U*_*r*_ = [*U* ^2^+(d*z*/d*t*)^2^]^1/2^, and the angle of attack due to oscillation is *α* = (d*z*/d*t*)/*U*. Therefore, drag *d*(*α*) and lift *l*(*α*) are as follows:

(1) $$\begin{array}{c} d\left(\alpha \right)=\frac{1}{2}\rho cU_{r}^{2}c_{d}\left(\alpha \right),\\ l\left(\alpha \right)=\frac{1}{2}\rho cU_{r}^{2}c_{l}\left(\alpha \right). \end{array}$$

In these expressions, *ρ* stands for the fluid density, *c* for a characteristic length normal to incident flow, *c*_*d*_ for the drag coefficient, and *c*_*l*_ for the lift coefficient. The projection of those forces in *z*-axis direction is *f*_*z*_(*α*) = −*d*(*α*)sin*α* − *l*(*α*)cos⁡*α*. On the other hand, the equation of the movement of the body, is

(2) $$\begin{array}{c} m\left[\frac{{\text{d}}^{2}z}{\text{d}t^{2}}+2\zeta \omega_{n}\frac{\text{d}z}{\text{d}t}+\omega_{n}^{2}z\right]=f_{z}\left(\alpha \right). \end{array}$$

Considering the movement to be quasisteady and assuming the angles of attack due to oscillation to be small enough (*α* ≪ 1), from the expression (2), the Glauert-Den Hartog criterion for galloping instability is derived. In effect, if the aerodynamic force (proportional in this case to d*z*/d*t*) is considered as a contribution to the total damping of the system (aerodynamic damping), the total damping coefficient is

(3) $$\begin{array}{c} \zeta_{T}=\zeta +{\frac{\rho Uc}{4m\omega_{n}}\left(\frac{\text{d}c_{l}}{\text{d}\alpha }+c_{d}\right)|}_{\alpha =0}, \end{array}$$

and therefore the oscillation will be stable if *ζ*_*T*_ > 0 and unstable if *ζ*_*T*_ < 0. As the mechanical damping *ζ* is generally positive, instability will only occur if (d*c*_*l*_/d*α* + *c*_*d*_) < 0, expression known as Glauert-Den Hartog criterion, which is a necessary condition for galloping instability. The sufficient condition is *ζ*_*T*_ < 0, or, according to (3):

(4) $$\begin{array}{c} {\left(\frac{\text{d}c_{l}}{\text{d}\alpha }+c_{d}\right)|}_{\alpha =0}<-\frac{4m\zeta \omega_{n}}{\rho Uc}. \end{array}$$

Later, in a number of papers, Novak [3, 4] extended the analysis of transverse galloping to the three-dimensional case. Other researchers have investigated other interesting phenomena like the influence of turbulence [6–10] or the hysteresis phenomenon [11, 12] in transverse galloping. In the last decades, besides theoretical work, large efforts have been devoted to experimentally study the galloping features of many bodies having different cross-sections. Although most of the effort in galloping oscillation research has been concentrated in bodies with square or rectangular cross-sections, prismatic bodies with other cross-sectional shapes have been also considered [13–18].

In the last years some research on galloping has been carried out at IDR/UPM because of the above-mentioned interest on the use of large louvers for sun-shading in building façades, amongst other applications. A systematic parametric analysis of simple cross-section two-dimensional bodies has been accomplished. The geometries analysed up to now are isosceles triangular cross-sections (the varying parameter being the main vertex angle, *β* [19–21] and ellipses [22], as well as biconvex and rhomboidal cross-sections [23] and the varying parameter in these last cases being the relative thickness of the cross-section, *τ*. In all cases, the unstable regions in the *β*-*α* plane (isosceles triangles) and in the *τ*-*α* plane (elliptical, biconvex, and rhomboidal bodies), where *α* stands for the wind angle of incidence, were determined).

Z-shaped cross-sections are not rare in modern architecture. Sun-shading louvers are examples where Z-shaped cross-sections may be found in slender structures susceptible to gallop. This type of Z-shaped cross-sections has also been experimentally analysed [24].

The validity of the numerical methods in the study of this kind of problems would have an important added value, since it would allow for predicting the aerodynamic properties of different shapes and profiles without performing the more expensive experimental tests. However, most of the numerical studies encountered are mainly focused on understanding the physical mechanism involved in flow separation of bluff bodies, such as circular, rectangular, square, and trapezoidal cylinders in a particular configuration. In this line, different high-fidelity numerical simulations have been performed using direct numerical simulation (DNS), large Eddy simulation (LES), Reynolds-averaged Navier-Stokes (RANS), or URANS models. Examples of these studies on cylinders of rectangular sections are provided by Tamura et al. [25], Hayashi and Ohya [26], and Rodi [27], in computing the mean aerodynamic coefficients using DNS. Hirano et al. [28] performed an LES simulation of the flow showing good accuracy, compared to the experimental results, for mean drag, lift, and the Strouhal number, or Oka and Ishihara [29], who also include an accurate method for estimating the aerodynamic coefficients using a systematic elongation of the spanwise length. Several RANS computations can be found in Bosch and Rodi [30] using the *k*-*ε* turbulence model and Bao et al. [31] who tried to use one-equation models such as Spalart-Allmaras. RANS models provide considerable saving in computational effort but normally have problems to get accuracy in flows with high pressure gradients.

Apart from the square and circular cross-sections, trapezoidal and triangular sections have also been analysed with details by several researchers: Lee [32], Cheng and Liu [33], and Oka and Ishihara [29] and Bao et al. [31] have studied the developing of the recirculation region vortex shedding phenomena and its interaction with the separated shear layer. These detailed works draw conclusions about the variation of the Strouhal number with the Reynolds number and aspect ratio.

Currently, these studies have been focused on the prediction of drag, lift, Strouhal number, and flow features in the downstream region of the profile for one particular configuration, showing that precise numerical computations can provide valuable information of the flow solutions in good agreement with experimental data. However, a thorough analysis of galloping stability requires the study of a large number of parameters (geometrical and physical), which makes previous computations, though accurate, nonpractical. In this line, Robertson et al. [34] made a numerical study of rotational and transverse galloping rectangular bodies using two-dimensional spectral high-order methods. Very good qualitative results were obtained showing the possibility to use numerical methods in the prediction and discussion of this problem. The work was performed at very low Reynolds numbers (250) far beyond current industrial configurations where turbulence effects can be of importance and although the separation point is almost independent of the Reynolds number in rectangular structures, it is not so for the reattachment length and base pressure.

To the authors' knowledge, a complete and critical study of the application of current “state of the art” numerical methods to the analysis of galloping instabilities at high Reynolds numbers has not been performed yet.

**Figure 1:**
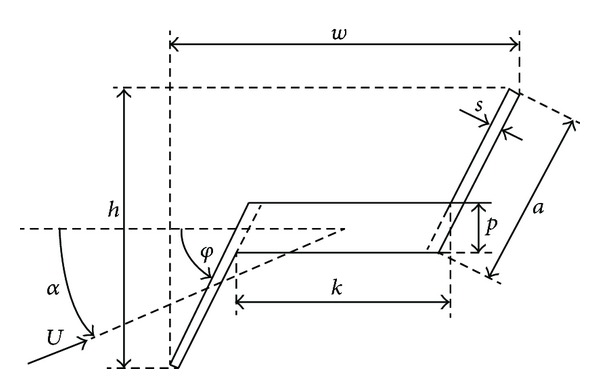
Z-shaped profile parameters.

In this paper, the transverse galloping characteristics of two-dimensional bodies having Z-shaped cross-sections (Figure 1) are analysed numerically. The overall aerodynamic forces and pressure distributions on the body walls as long as the stability maps are computed and compared with referenced experimental test cases. The study is a critical demonstration of the feasibility of using standard industrial solvers in the prediction of galloping. So, contrary to other authors, we are more interested in global results and computational efficiency than the detailed analysis of one particular configuration. After a first evaluation of different models, the numerical solution is solved with a 2D RANS-SST turbulence model which provides a good compromise between accuracy and computational effort. Detailed analysis of some critical configurations where numerical and experimental results show some disagreement is also included. In order to clarify the influence of the geometry on the transverse galloping, fourteen different Z-shaped cross-sections have been considered.

The paper is organized as follows. In Section 2, after the definition of the geometry and flow condition of the problem, a short introduction of the DLR TAU Code is used in the computations and a critical evaluation of different turbulence models and grid sensitivity are performed.  Section 3 shows the aerodynamic coefficients, stability maps, and pressure distributions of the whole range of configurations with different aspect ratios. A comparison between RANS and URANS for some critical configuration is also included. Finally, Section 4 extracts the conclusions from the study.

## 2. Numerical Analysis and Validation Tests

### 2.1. Problem Description

**Figure 2:**
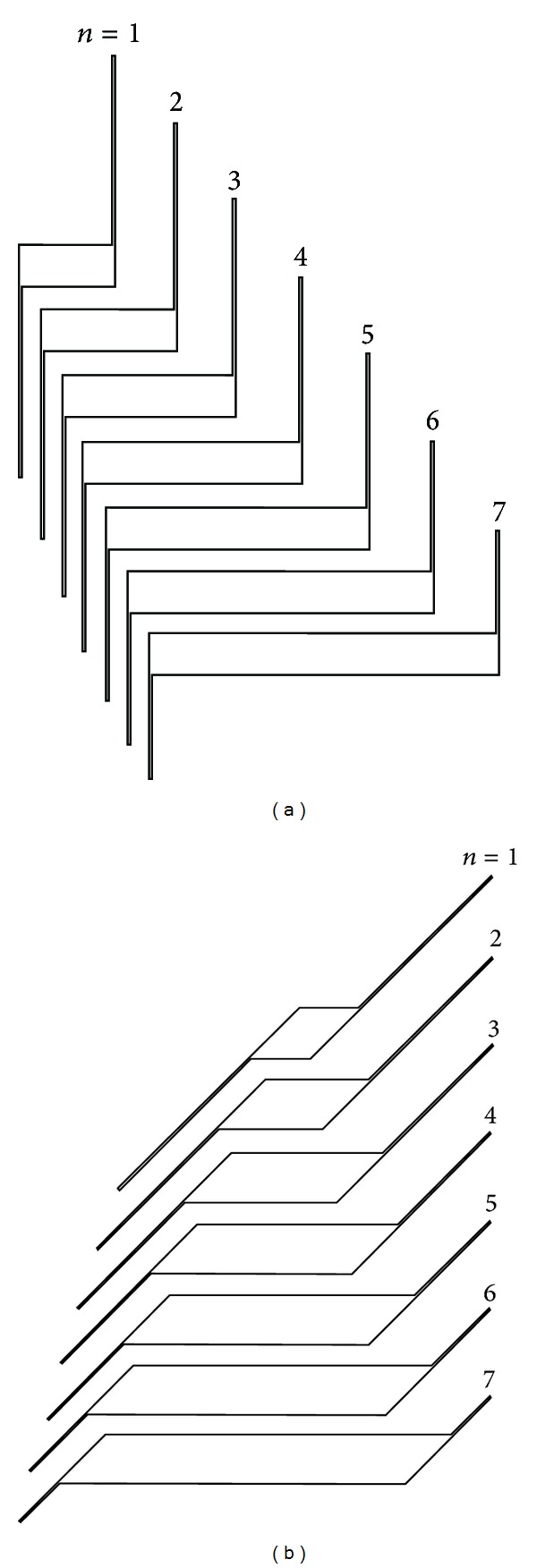
Set of tested louver Z-shaped profiles with *φ* = 90° (a) and *φ* = 45° (b) with *n* = 1, 2,…, 7.

The Z-shaped louvers are made out a thick central body (web) with two thin plates attached to its extremes (wings). The geometry of the Z-shaped louvers is defined by some geometrical parameters shown in Figure 1. All the parameters are in function of the chord of the slats, *c*, the slat angle *φ*, and *n*. The angle *φ* is defined by the angle between the central body and the wings; *n* is an integer used to classify the different the aspect ratios studied. Two different families of louvers, identified by the angle *φ* and for each one of them different geometries, were considered, namely *φ* = 90°, with *n* = 1, 2,…, 7, and *φ* = 45°, with *n* = 1, 2,…, 7. The whole set of tested louver shapes is shown in Figure 2.

These configurations have been experimentally studied at IDR/UPM, Alonso et al. [24]. The test facility is a two-dimensional open circuit wind tunnel where the test chamber dimensions are 0.15 m width, 0.90 m high, and 1.20 m long. The aerodynamic loads were measured with a six-component strain-gauge balance. Additional details of the wind tunnel chamber and the experimental results can be found in [24]. The flow conditions were an inlet velocity of 25 m/s, a Reynolds number of 1.7 · 10^5^ with the chord, *c* = 0.1 m, as reference length, and 4% of turbulence intensity. The angles of attack used for the numerical study vary from 0° to 180° with increments of 5°.

The parameters used in the present study are defined to match the experimental results: the Reynolds number, Re, is defined on the basis of the free stream velocity and the chord of the slats as reference length, giving a value of 1.7 · 10^5^. The drag and lift coefficients are defined as *c*_*d*_ = 2*d*/*ρU*_*∞*_^2^ *c*, *c*_*l*_ = 2*l*/*ρU*_*∞*_^2^ *c*, respectively; *d* and *l* are the drag an lift forces acting on the Z-profile, respectively, and *ρ* is the flow density. The pressure coefficient is defined as *c*_*p*_ = 2(*p* − *p*_*∞*_)/*ρU*_*∞*_^2^, with *p*_*∞*_ being the pressure at the far field.

The case matrix of the numerical study is given in Table 1.

### 2.2. DLR TAU Solver

The industrial DLR TAU Code [35] is used in the computations. The DLR TAU Code solves the compressible Reynolds-averaged Navier-Stokes (RANS) equations on unstructured hybrid grids employing a second-order finite volume discretization. The solver uses an edge-based dual-cell approach, that is, a vertex-centred scheme. The inviscid terms are computed employing either a second-order central scheme with the standard Jameson-Schmidt-Turkel (JST) numerical dissipation model or a variety of upwind schemes using linear reconstruction for second-order accuracy and a scalar or matrix artificial dissipation. Viscous and turbulent terms are discretized with a central second-order scheme.

The turbulence models implemented within the TAU code include linear as well as nonlinear eddy viscosity models spanning to both one and two equation model families.

Two different turbulence models have been tested in the computations: the Spalart-Allmaras original (SAO) [36], yielding highly satisfactory results for a wide range of applications while being numerically robust, and the two equation standard *k*-*ω* model and its variation, the SST (shear-stress-transport) *k*-*ω* model [37], which combines several desirable elements of *k*-*ω* and *k*-*ε* models. The SST (shear-stress-transport) *k*-*ω* model uses Wilcox's *k*-*ω* with a good behaviour in modelling the near solid walls and the standard *k*-*ε* model, appropriate for near boundary layer edges and free-shear layers. This turbulence model is appropriate for adverse pressure gradients and separating flow.

### 2.3. Mesh Validation

**Figure 3:**
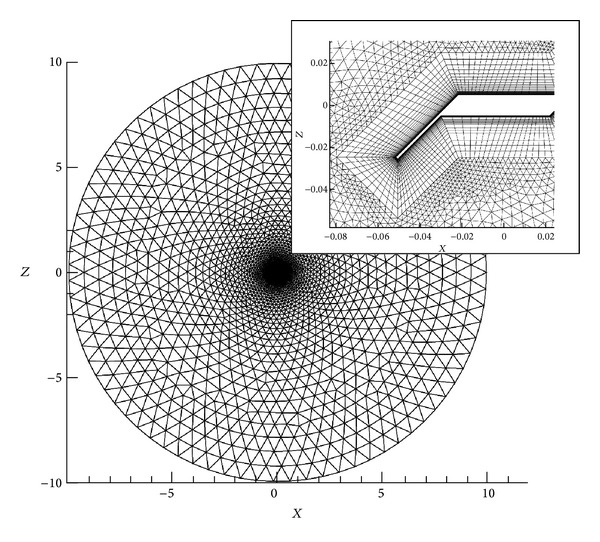
Computational circular domain and hybrid mesh used in the present study.

Figure 3 shows the computational domain and a sketch of a typical mesh used in the present study. A 2D hybrid mesh allows creating a good resolution boundary layer zone with quadrilateral and triangular elements for the rest of the domain. A circular domain with a radius of 100-chords (100*c*) is employed, which ensures that far field boundary condition does not affect the numerical results.

**Figure 4:**
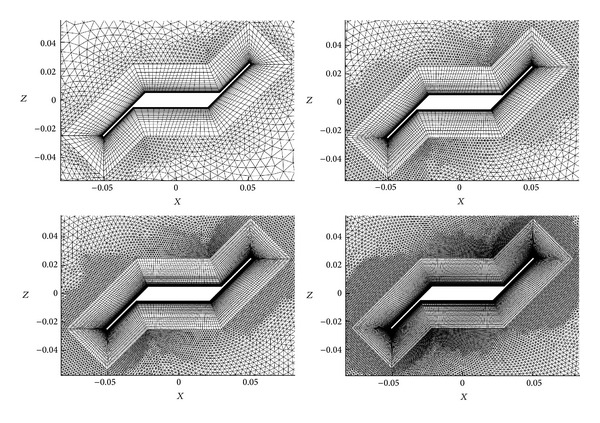
Validation grids with different refinement (coarse, medium, fine, and extrafine).

Four different grids (see Table 2) of the same configuration (*φ* = 45° and *n* = 5, angle of attack *α* = 60°) with different densities have been created to check the mesh convergence of the numerical results. The four meshes (Figure 4) have been solved with the Spalart-Allmaras model turbulence.

No-slip boundary condition is imposed at the wall of the profile. To solve correctly the boundary conditions, it is important to define the *y* ^+^ carefully, (*y* ^+^ = *u* ^+^Δ*y*/*ν*); *y* ^+^ depends on the wall normal direction near the surface of the profile (Δ*y*), the viscosity (*ν*), and the friction velocity (*u* ^+^). In this study, Δ*y* has been fixed to 5 · 10^−5^ m, which gives a value of *y* ^+^ around the unity for all the cases.

Local time step, multigrid acceleration, and LU-SGS semi-implicit smoother, with a Courant-Friedrich-Levy number, CFL = 1.5, are used in the computations of the steady states. Moreover, the Jameson-Schmidt-Turkel (JST) numerical dissipation model has been used with *k*_2_ and *k*_4_ coefficients fixed in 2/3 and 1/64, respectively, (values by default in DLR TAU Code). These values have been proven to give a good numerical stability of the solution.

The DLR-TAU solver uses a preconditioner for the low-mach number problem. This preconditioner reduces the stiffness of the problem by rendering the eigenvalues of the Jacobian matrix of the same magnitude, acting on the temporal derivative of the unknowns. A modification of the well-known Choi-Merkle and Turkel preconditioner scheme is implemented in TAU (see Turkel et al. [38] for details).

The preconditioner is only activated for steady solutions. In case of unsteady problems, it should be deactivated to recover the temporal accuracy. A lower time step than usual is considered to reduce numerical errors associated with the low-mach number problem.

The simulations have been computed in an 8 processors multicore cluster. Each node is an Intel(R) Xeon(R) CPU E5620 @ 2.40 GHz with 24 Gb of RAM. The computational time for each simulation is shown in Table 2.

**Figure 5:**
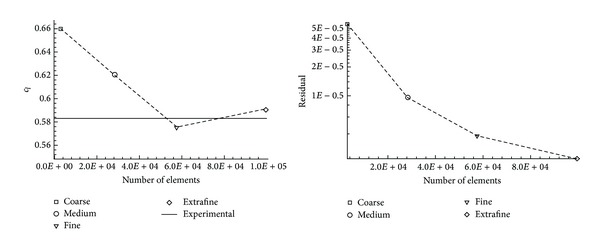
Convergence of the aerodynamic coefficients and residual in function of the number of elements.

The aerodynamic coefficients as a function of the number of elements, obtained with these four validation meshes, are shown in Figure 5. The numerical results converge to the experimental value for this configuration. With the current results, the fine mesh (see Table 2), which guarantee a good compromise between mesh independence and the computational effort, has been chosen to perform the battery of the simulations of this study.

### 2.4. Turbulence Model Validation

In order to compare the effect of the turbulence model, a complete lift and drag curves are obtained for the configuration *φ* = 45° and *n* = 5. The angle of attack ranged from 0° to 180° with an increment of 2°, making a total of 46 angles of attack computed.

As previously mentioned, the Navier-Stokes equations have been solved with the Spalart-Allmaras original (SAO) and the *k*-*ω* SST (shear-stress-transport) turbulence models. In addition to those RANS computations, the problem has also been solved with the Euler equations.

**Figure 6:**
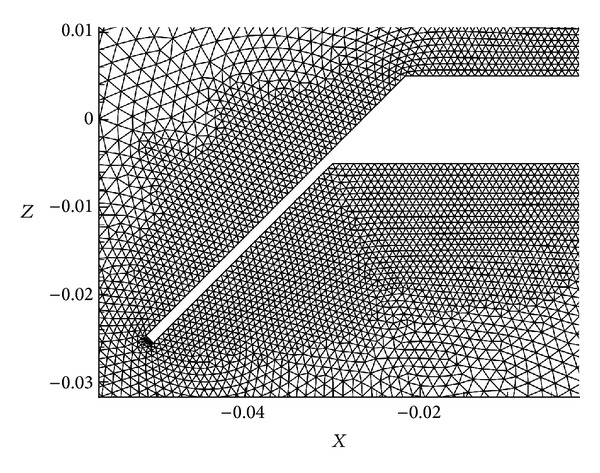
Euler mesh without refinement for the boundary layer.

The reason to solve the problem with Euler equations is the fact that, in this kind of profiles, the detachment of the flow is geometry induced (and almost independent of the Reynolds number), instead of a pressure induced separation, which requires a better modelling of the boundary layer. A mesh (Figure 6) without refinement for the boundary layer, created with 40604 triangle elements, has been used in this computation. The Euler solution has the advantage of requiring coarse meshes, since the boundary layer is not solved, which obviously will affect the computational cost, and taking into account the large number of simulations to carry out, the choice of the turbulence model is balanced between the accuracy of the results and the computational time. The aim of this comparison is to check the capacity of the turbulence model in obtaining the base pressure, in the vortex detached zone behind the profile, which have a strong impact on the final forces.

Figure 8 shows a comparison between the three simulations. It is observed that the Euler results underpredict the stall area, which confirms the fact that even in this kind of configurations it is necessary to account for the viscosity in the prediction of the detachment. Therefore, it is necessary to use Navier-Stokes with turbulence model even if it means an increase in the computational time as observed in Table 3.

An additional analysis of the simulations shows good agreement with the experimental results. However, there are two areas where the turbulence models and the experiments show largest discrepancies (see Figure 8). The *c*_*l*max⁡_ and the *c*_*d*_ at high angle of attack zones are better predicted by the *k*-*ω* SST. The *k*-*ω* SST model estimates, with more accuracy, the base pressure value, giving overall better results. Finally, the *k*-*ω* SST has been selected to solve all the configurations. A more detailed study of these two areas is shown in Section 3.2.

As an additional validation, it has been checked the possibility that the wind tunnel blockage could substantially modify the numerical results. To this end, the dimensions of the wind tunnel test chamber which is 0.15 m width, 0.90 m high, and 1.20 m long, have been reproduced. In this case, different meshes for the following angles of attack: 0°, 20°, 40°, 60°, 80°, 100°, 120°, 140°, 160°, and 180° have been created.

**Figure 7:**
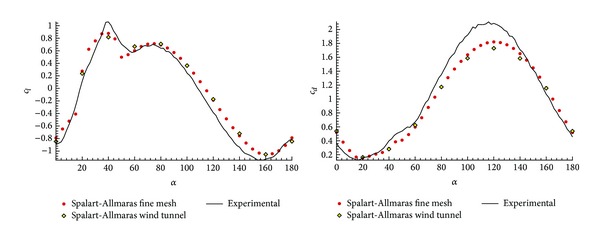
Checking the possibility of wind tunnel blockage simulation in the experimental results with Spalart-Allmaras.

**Figure 8:**
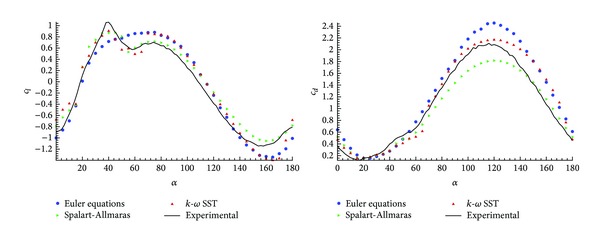
Validation model turbulence in medium grid *n* = 5,  *φ* = 45° with Euler equations, Spalart-Allmaras, and *k*-*ω* SST turbulence model.

The results in Figure 7 show no difference with and without tunnel blockage consideration. This conclusion allows using the mesh with circle domain for all the rest of configurations, which facilitates the numerical study in terms of grid generation.

## 3. Numerical Results for Galloping Stability Analysis

After completion of the problem definition, mesh, and turbulence model validation, the simulation of the galloping instability maps was computed using the fine mesh and a *k*-*ω* SST RANS turbulence model.

The computations are “steady,” which means that some flow unsteady features that can appear in the solution are numerically damped. We will see in our analysis that, in most of the cases, the numerical steady solutions give enough resolution for standard industrial problems; however, unsteady detailed computations can improve the global estimations in certain configurations where, as we will see, the unsteady features are more important.

As previously mentioned, the angle of attack is varied from 0° to 180° with an increment of 2°, making a total of 46 angles of attack for each configuration.

### 3.1. Aerodynamic Coefficients

**Figure 9:**
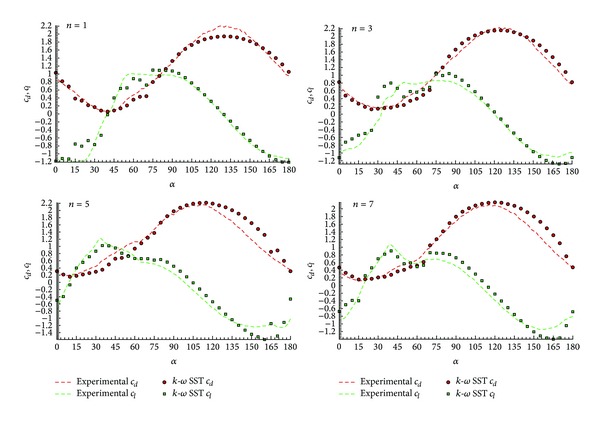
Lift and drag coefficient integral over the surface profile of each configuration (*n* = 1,3, 5 and 7), for *α* = 45°.

**Figure 10:**
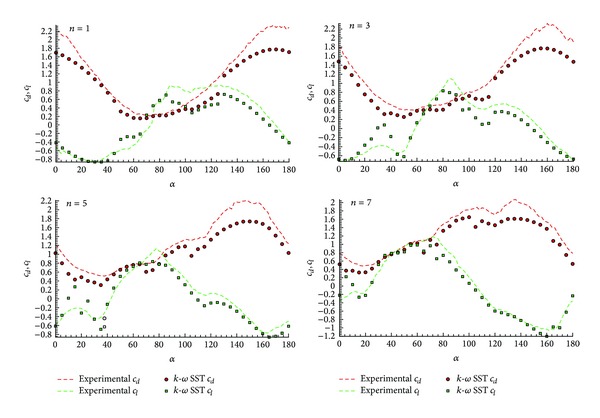
Lift and drag coefficient integrate over the surface profile of each configuration (*n* = 1,3, 5 and 7), for *α* = 90°.

As a preliminary result, Figure 9 shows the lift and drag coefficient integrated over the surface profile for four representative configurations (*n* = 1, *n* = 3, *n* = 5, and *n* = 7) of the *φ* = 45° family profiles. The numerical results agree well with the experimental values even in the critical *c*_*l*max⁡_ and in the *c*_*d*_ values at high angles of attack regions. This is not the case for the *φ* = 90° family profiles. In Figure 10, the lift and drag coefficients for configurations *n* = 1, 3, 5, and 7, for *φ* = 90°, are shown. In these cases, some differences in the areas of *c*_*l*max⁡_ and the *c*_*d*_ values at high angles of attack are numerically underestimated.

**Figure 11:**
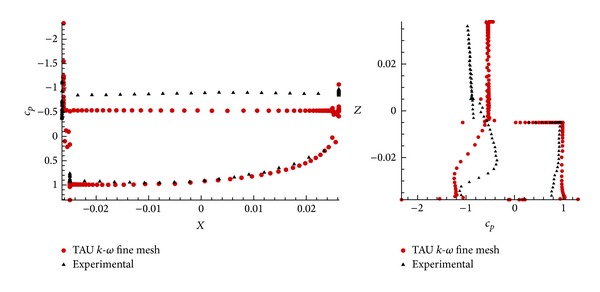
Value of pressure coefficient in function of the both coordinates, *X* and *Z*, for *α* = 75° in configuration *n* = 4; *φ* = 90°.

Comparison of the pressure distribution at *φ* = 90° and *n* = 4 provides an explanation of the reasons of these discrepancies.  Figure 11 sketches the values of *c*_*p*_ as a function of the coordinates, *X* and *Z*, at *α* = 75°, in the region of *c*_*l*max⁡_. It is shown that on the upstream part of the profile, numerical and experimental results agree accordingly but, as expected, in the downstream region, the detached flow shows differences between the base pressures. These differences are the cause of the discrepancies between the aerodynamic forces.

The main reason of this inaccuracy is due to the strong unsteady flow which appears in that region, and it is not properly solved by a steady RANS solver. A better resolution should be obtained by performing an unsteady simulation with a time step small enough to solve these structures. As we will see in Section 3.2, these structures have a strong impact on the evaluation of the global coefficients.

**Figure 12:**
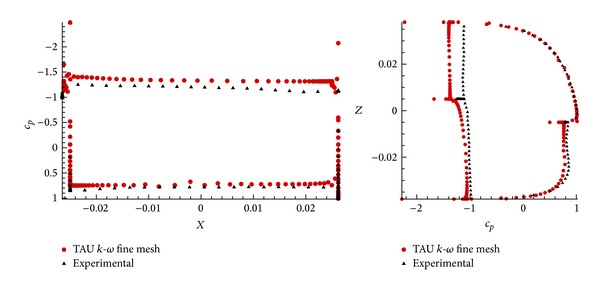
Value of pressure coefficient in function of the both coordinates, *X* and *Z*, for *α* = 100°  in configuration *n* = 4; *φ* = 90°.

For demonstration purposes, Figure 12 also shows the *c*_*p*_ values as a function of the coordinates, *X* and *Z*, in this case at *α* = 100°, where a good agreement between numerical and experimental lift is obtained. As expected, the numerical and experimental pressure values are in good consonance.

However, we will see in Section 3.3 that these discrepancies do not have a strong influence in the stability maps, since they are more defined by the global behavior of the coefficients than the detailed solutions.

### 3.2. Unsteady Simulation (URANS)

The URANS numerical method is used to improve the prediction and explain the discrepancies in the two areas where a lack of accuracy has appeared: the *c*_*l*max⁡_ and the *c*_*d*_ values at high angles of attack. It is important to see if, by improving the numerical resolution of the solution, it is possible to obtain additional details of the downstream flow structures which eventually produce changes on the average pressure distribution over the profile.

The URANS computations use a dual-time stepping scheme. A Backward difference formula (BDF) is used to discretize the global time and a fourth-order Runge Kutta with multigrid acceleration scheme being employed to obtain a steady state in the fictitious pseudotime. Taking into account that the frequency of the oscillation is unknown a priori, in order to define the global time-step of the computations, first, a characteristic time scale is defined based on the convective velocity and the length of the profile (*t*_*c*_ ≈ *L*/*U*_*∞*_). The first estimation of the time-step is taken as Δ*t*_*c*_ = *t*_*c*_/100. With this time step, an unsteady oscillation of 100 Hz frequency is computed. Finally, a more detailed simulation has been computed taking 100 steps per cycle, giving a global time step of (with the CFL = 1,) Δ*t*_*c*_ = 10^−5^.

The unsteady computation is initialized with the steady solution and evolved until statistical convergence, which requires a total number of 6 · 10^4^ time steps to get a simulation time of 0.6 s. The mean values of this unsteady simulation are compared with the results obtained with the RANS solver.

**Figure 13:**
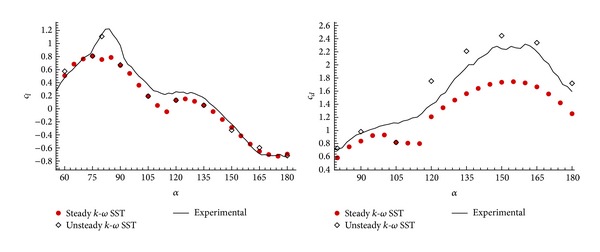
Aerodynamic coefficients comparison between the RANS and URANS simulation with the experimental results in configuration *n* = 4; *φ* = 90°.

**Figure 14:**
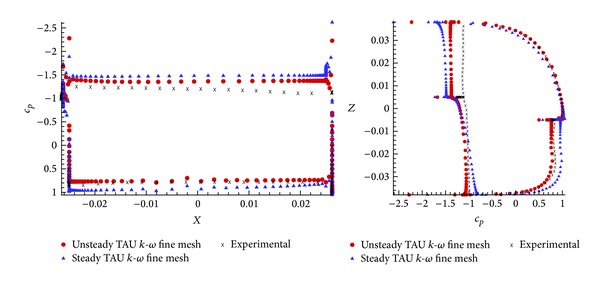
Pressure coefficient comparison between RANS and URANS at angle of attack *α* = 165° configuration *n* = 4; *φ* = 90°.

Figure 13 shows the comparison between the aerodynamic coefficients obtained with the RANS and URANS simulations and the experimental results. As expected, the *c*_*l*max⁡_ and the *c*_*d*_ values at high angles of attack have greatly improved compared to the experimental results. The rest of the angles of attack, where unsteady effects are negligible, keep the same accuracy of steady computations. By comparing the mean pressure distribution (Figure 14) at angle of attack *α* = 165°, it is observed how the URANS results modify the mean base pressure to provide a better accuracy compared to experimental data on *c*_*l*_ and *c*_*d*_. An additional improvement should be possible if complex numerical models, like LES or DES, are used, but at the cost of increasing even more the computational effort.

**Figure 15:**
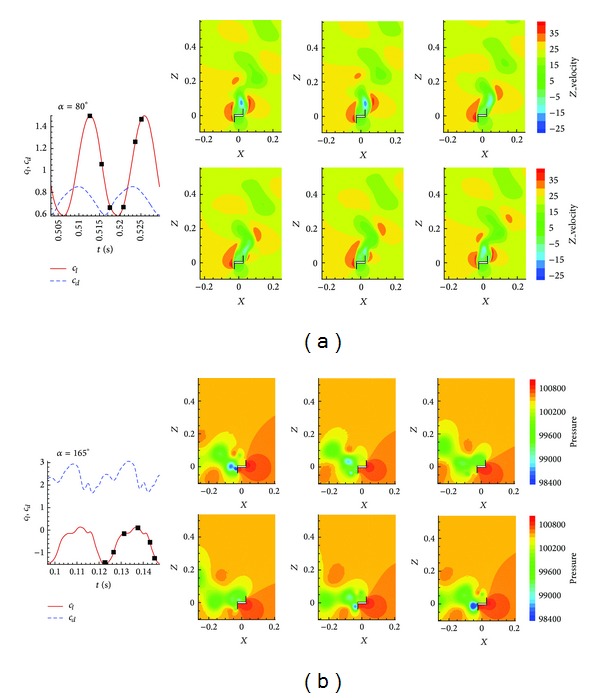
Pressure field of a period at angle of attack *α* = 80° (a) and 165° (b).

Finally, for demonstration purposes, Figure 15 reproduces the pressure contours of the numerical solution at different time steps in a temporal period of lift. The flow follows a typical von Kármán vortex street with a main detachment frequency of 76 Hz; the detachment appears in both wings of the profile alternately.

In terms of computational effort, Table 4 shows the results of *c*_*l*_ and *c*_*d*_ and the computational time for the angle of attack *α* = 80° between the steady and unsteady simulations. The unsteady simulation takes around 24 times the steady computational time; this is a difference to be taken into account.

### 3.3. Stability Maps

The stability parameter (*H* = d*c*_*l*_/d*α* + *c*_*d*_) following the Glauert-Den Hartog criterion and the numerical computations have been calculated with a central difference approximation for the first derivative. Despite the detailed numerical analysis which shows that for some angles of attack, better accuracy is obtained with unsteady simulations, in the computation of the stability maps only “industrial” steady solutions are considered for comparison.

**Figure 16:**
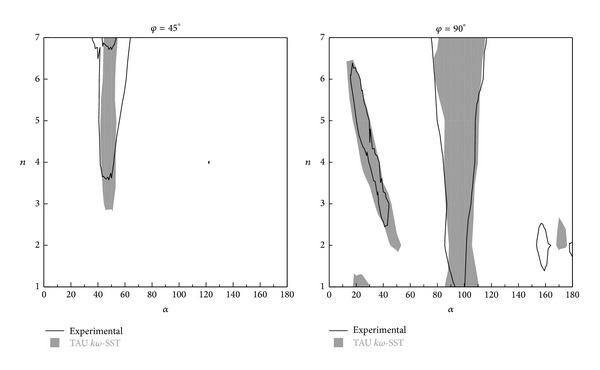
Stability map of *φ* = 45° and *φ* = 90° family profiles as a function of *n* and *α*.

In Figure 16, the numerical and experimental values of *H* as a function of the parameter *n* are compared for both configurations studied, *φ* = 45° and *φ* = 90°. Inside the shadow areas, the stability parameter (*H*) value is negative; in other words, the Glauert-Den Hartog criterion is satisfied and galloping instability appears. Consider

(5) $$\begin{array}{c} \varphi =45^{{}^{\circ}}. \end{array}$$

As it can be observed, a good agreement between the experimental and numerical results is obtained even for the critical regions of *c*_*l*max⁡_ and high angles of attack. Nonetheless, some differences are observed: in the family profiles *φ* = 45°, a good agreement in the area where the instability appears (in the range of angles of attack between *α* = 40° and *α* = 60°) is found. As depicted in Figure 9, this area corresponds to the region of maximum lift and close to the minimum drag, and it is related to the two-peak structure shown for higher values of *n*. Indeed, for lower values of *n*, the lift curve shows a large flat zone close to its maximum and galloping instability does not show up. For *n* = 3 or higher, a first peak of maximum lift appears around *α* = 40°, followed by a strong decay and a second maximum close to *α*~60°–70°. This effect is more accused as long as *n* is increased. It is precisely this structure which defines the galloping instability. At higher values of *n*~6-7, the two peaks separate giving place to a small lift flat region between them; this eventually can produce a stable area between these two branches. Although the global magnitudes are well predicted, this particular detail is not well captured by the numerical method, mainly because of the lack of resolution in the Δ*α* of the predicted lift curves. Consider

(6) $$\begin{array}{c} \varphi =90^{{}^{\circ}}. \end{array}$$

Despite some numerical inaccuracies observed in the numerical computations of this case, the instability regions of the steady numerical solutions are well captured and agree with the experimental results.

Two main regions of instability appear for this configuration: a region at low angles of attack (between *α* = 20° and *α* = 40°) for *n* values, ranging from 2 to 6, and a second area for with *α* = 90°–110° for all *n*. The first region is related to a peak of lift at around *α* = 20°. Although the numerical results are somehow inaccurate and this maximum lift is overpredicted (making necessary unsteady computation to improve the results), this inaccuracy has almost no effect on the instability regions, since the global behavior is well captured. The second region (*α* = 90°–110°) shows a similar behavior to the case *φ* = 45°. The presence of two maxima in the lift coefficient in a region of growing drag is the cause of galloping. The region is slightly wider for larger values *n*, effect which is well reproduced by the numerical and experimental data.

An additional and small region appears at high angles of attack (*α*~160°–170°). This region is predicted by both numerical and experimental results, but slightly shifted to lower *α* in the numerical results. The reason to that is the lack of numerical precision in this area. The problem has been discussed in the previous section, and unsteady numerical computations are able to improve these results.

Even with these differences, only with the numerical results we are able to design our geometry in terms of the stability region. Within the shadow region, the necessary condition for transverse galloping is satisfied, so this design geometry is appropriated to extract energy through movement caused by this phenomenon. Otherwise, if the goal is to avoid this phenomenon, the geometry will be designed with a configuration which is outside of the transverse galloping instability zone.

## 4. Conclusions 

The use of wind tunnel tests for the evaluation of the aerodynamic coefficients can be sometimes difficult and costly. This paper investigates the suitability of numerical models for the prediction of galloping instability of bodies with different cross-sections. A numerical method based on the DLR TAU Code has been used. The method has been validated, comparing the results with experimental measurements, for a cross-section which is very interesting from the civil construction point of view: the Z-profile. The Glauert-Den Hartog criterion has been used to determine the galloping regions of instability with respect to the incident wind angle of attack and cross-section geometrical parameters.

This paper reports the results obtained in a systematic analysis of the galloping instability, performed on a family of Z-shaped cross-sections. This profile is made out of a central web and two side wings. The analysis determines under which interval of angles of attacks and geometrical parameters, and this type of profiles become unstable. From the computed aerodynamic coefficients the *H*(*φ*, *α*, *n*) = d*c*_*l*_/d*α* + *c*_*d*_ functions have been determined and from them stability maps in the *n*-*α* plane have been plotted.

According to the results, the family of profiles with better performance against the apparition of transverse galloping instabilities is *φ* = 45°. Within this family, instabilities can still appear at angles of attack between 40° and 60°, contrary to profiles with smaller aspect ratio, for which this phenomenon does not appear. On the contrary, if the goal is to induce this phenomenon to extract energy from the movement [39], the suitable profiles are with *φ* = 90° because they are more prone to gallop.

Good accuracy in the prediction of the galloping instability with the numerical method (DLR TAU Code) has been obtained.

The methodology employed allows important savings in computational effort.

It has been observed that the RANS simulation is good enough to predict the necessary condition for the occurrence of the transverse galloping phenomenon; that is, the Glauert-Den Hartog criterion is satisfied. However, better accuracy can be obtained (at a higher computational costs) in the area where some discrepancies still appear.

## Figures and Tables

**Table 1:** Case matrix of the numerical study.

	*n* = 1	*n* = 2	*n* = 3	*n* = 4	*n* = 5	*n* = 6	*n* = 7
*φ* = 45°	*✓*	*✓*	*✓*	*✓*	*✓*	*✓*	*✓*
*φ* = 90°	*✓*	*✓*	*✓*	*✓*	*✓*	*✓*	*✓*

**Table 2:** Characteristics of validation grids.

α = 60°	Number of elements	Number of nodes	Residual	Conver. iterations	Comp. time (min)
Coarse	12923	16642	5.6*e* − 5	50000	4.6
Medium	28397	35632	9.5*e* − 6	100000	11.6
Fine	57408	70276	3.8*e* − 6	200000	67.2
Extra fine	99562	119552	2.2*e* − 6	300000	143.4

**Table 3:** Comparison between the computational cost of Euler and RANS calculations for *α* = 60°. Similar values are obtained in other configurations.

	Number of elements	Residual	Conver. iterations	Comp. time (min)
Euler	12923	3.5*e* − 4	200000	16.0
Fine-SA	57408	3.8*e* − 6	200000	67.2
Fine-*k*-*ω* SST	57408	1.0*e* − 8	200000	77.0

**Table 4:** Comparison between RANS, URANS, and experimental values of *c*_*l*_, *c*_*d*_ and the computational time for the angle of attack *α* = 80°.

*α* = 80°	*c*_*l*_	*c*_*d*_	Comp. time (h)
Experimental	1.14	0.7155	—
Steady	0.755	0.584	1.1
Unsteady	1.108	0.728	23.8

## References

[1] den Hartog JP (1956). *Mechanical Vibrations*.

[2] Parkinson G, Smith J (1964). The square prism as an aeroelastic non-linear oscillator. *The Quarterly Journal of Mechanics and Applied Mathematics*.

[3] Novak M (1969). Aeroelastic galloping of prismatic bodies. *Journal of Engineering Mechanics Division*.

[4] Novak M (1972). Galloping oscillations of prismatic structures. *Journal of Engineering Mechanics Division*.

[5] Simiu E, Scanlan RH (1996). *Wind Effects on Structures. Fundamentals and Applications to Design*.

[6] Novak M, Tanaka H (1974). Effect of turbulence on galloping instability. *Journal of the Engineering Mechanics Division*.

[7] Li QS, Fang JQ, Jeary AP (1998). Evaluation of 2D coupled galloping oscillations of slender structures. *Computers and Structures*.

[8] Ziller C, Ruscheweyh H (1997). A new approach for determining the onset velocity of galloping instability taking into account the nonlinearity of the aerodynamic damping characteristic. *Journal of Wind Engineering and Industrial Aerodynamics*.

[9] Hémon P, Santi F, Schnoerringer B, Wojciechowski J (2001). Influence of free-stream turbulence on the movement-induced vibrations of an elongated rectangular cylinder in cross-flow. *Journal of Wind Engineering and Industrial Aerodynamics*.

[10] Hémon P, Santi F (2002). On the aeroelastic behaviour of rectangular cylinders in cross-flow. *Journal of Fluids and Structures*.

[11] Luo SC, Chew YT, Ng YT (2003). Hysteresis phenomenon in the galloping oscillation of a square cylinder. *Journal of Fluids and Structures*.

[12] Barrero-Gil A, Sanz-Andrés A, Alonso G (2009). Hysteresis in transverse galloping: the role of the inflection points. *Journal of Fluids and Structures*.

[13] Ruscheweyh H, Hortmanns M, Schnakenberg C (1996). Vortex-excited vibrations and galloping of slender elements. *Journal of Wind Engineering and Industrial Aerodynamics*.

[14] Kawai H (1998). Effect of corner modifications on aeroelastic instabilities of tall buildings. *Journal of Wind Engineering and Industrial Aerodynamics*.

[15] Luo SC, Chew YT, Lee TS, Yazdani MG (1998). Stability to translational galloping vibration of cylinders at different mean angles of attack. *Journal of Sound and Vibration*.

[16] Richardson AS (1988). Predicting galloping amplitudes. *ASCE Journal of Engineering Mechanics*.

[17] Blevins RD (1990). *Flow-Induced Vibrations*.

[18] Naudascher E, Rockwell D (1994). *Flow-Induced Vibrations: An Engineering Guide*.

[19] Alonso G, Meseguer J, Pérez-Grande I (2005). Galloping instabilities of two-dimensional triangular cross-section bodies. *Experiments in Fluids*.

[20] Alonso G, Meseguer J, Pérez-Grande I (2007). Galloping stability of triangular cross-sectional bodies: a systematic approach. *Journal of Wind Engineering and Industrial Aerodynamics*.

[21] Alonso G, Meseguer J (2006). A parametric study of the galloping stability of two-dimensional triangular cross-section bodies. *Journal of Wind Engineering & Industrial Aerodynamics*.

[22] Alonso G, Meseguer J, Sanz-Andrés A, Valero E (2010). On the galloping instability of two-dimensional bodies having elliptical cross-sections. *Journal of Wind Engineering and Industrial Aerodynamics*.

[23] Alonso G, Valero E, Meseguer J (2009). An analysis on the dependence on cross section geometry of galloping stability of two-dimensional bodies having either biconvex or rhomboidal cross sections. *European Journal of Mechanics B*.

[24] Alonso G, Pérez-Grande I, Meseguer J Galloping instabilities of Z-shaped shading louvers.

[25] Tamura T, Ohta I, Kuwahara K (1990). On the reliability of two-dimensional simulation for unsteady flows around a cylinder-type structure. *Journal of Wind Engineering and Industrial Aerodynamics*.

[26] Hayashi K, Ohya Y A numerical study of the flow around a rectangular cylinder with critical depth.

[27] Rodi W (1997). Comparison of LES and RANS calculations of the flow around bluff bodies. *Journal of Wind Engineering and Industrial Aerodynamics*.

[28] Hirano H, Maruoka A, Watanabe S (2002). Calculations of aerodynamic properties of rectangular cylinder with slenderness ratio of 2:1 under various angles of attack. *Journal of Structural Engineering A*.

[29] Oka S, Ishihara T (2009). Numerical study of aerodynamic characteristics of a square prism in a uniform flow. *Journal of Wind Engineering and Industrial Aerodynamics*.

[30] Bosch G, Rodi W (1998). Simulation of vortex shedding past a square cylinder with different turbulence models. *International Journal Numerical Methods in Fluids*.

[31] Bao Y, Zhou D, Huang C, Wu Q, Chen X (2011). Numerical prediction of aerodynamic characteristics of prismatic cylinder by finite element method with Spalart-Allmaras turbulence model. *Computers and Structures*.

[32] Lee TS (1998). Early stages of an impulsively started unsteady laminar flow past tapered trapezoidal cylinders. *International Journal for Numerical Methods in Fluids*.

[33] Cheng M, Liu GR (2000). Effects of afterbody shape on flow around prismatic cylinders. *Journal of Wind Engineering & Industrial Aerodynamics*.

[34] Robertson I, Li L, Sherwin SJ, Bearman PW (2003). A numerical study of rotational and transverse galloping rectangular bodies. *Journal of Fluids and Structures*.

[35] DLR Institute

[36] Spalart PR, Allmaras SR (1992). A one-equation turbulence model for aerodynamic flows. *AIAA Paper*.

[37] Menter FR (1993). Zonal two equation k-*ω* turbulence models for aerodynamic flows. *AIAA Paper*.

[38] Turkel E, Radespiel R, Kroll N (1997). Assessment of preconditioning methods for multidimensional aerodynamics. *Computers and Fluids*.

[39] Alonso G, Sanz-Andrés A, Barrero-Gil A (2010). Energy harvesting from transverse galloping. *Journal of Sound and Vibration*.

